# Efficacy of Sorafenib Combined with Interventional Therapy on Primary Liver Cancer Patients and Its Effect on Serum AFP, VEGF, and GGT

**DOI:** 10.1155/2021/9120265

**Published:** 2021-08-11

**Authors:** Ying Jia, Yufei Xing, Meitian Yang

**Affiliations:** ^1^Department of Laboratory, Tianjin Jianhua Hospital, Tianjin 300122, China; ^2^Department of Laboratory, The Second People' Hospital of Dongying, Dongying 257335, China

## Abstract

**Objective:**

To explore the efficacy of sorafenib combined with interventional therapy on primary liver cancer (PLC) patients and its effect on serum AFP, VEGF, and GGT.

**Methods:**

120 PLC patients admitted to our hospital from January 2016 to January 2020 were selected as the research object and divided into group A and group B according to the admission order, with 60 cases each. Interventional therapy was performed to both groups, and sorafenib was given to group A additionally to compare their treatment effect, survival, adverse reaction rate (ARR), and serum AFP, VEGF, and GGT levels.

**Results:**

After treatment, group A obtained significantly higher objective remission rate (ORR) and disease control rate (DCR) (*p* < 0.05), higher one-year survival rate and two-year survival rate (*p* < 0.05), lower ARR of skin reactions, gastrointestinal reactions, hepatorenal reactions, and hyperbilirubinemia (*p* < 0.05), and lower serum AFP, VEGF, and GGT levels (*p* < 0.001).

**Conclusion:**

The combination of sorafenib and interventional therapy can inhibit the growth and migration of PLC, improve the immune function, prolong the survival period of patients, and lower ARR, so it should be promoted in practice.

## 1. Introduction

Primary liver cancer (PLC) is one of the most common malignant tumors in the clinic, and its lethality rate ranks the third place in all malignant tumors after lung cancer and gastric cancer with more than a million patients dying each year due to the disease. PLC is characterized by insidious onset and slow tumor growth, so most patients have missed the optimal surgical time when being diagnosed and can no longer receive radical treatment [1–3]. For PLC patients in advanced or middle advanced stages, local nonsurgical treatment options are the preferred measures to control the further spread of cancer cells, and interventional treatment is the most commonly used in the clinic, which is able to block the blood supply to the hepatic artery, make the cancer cells ischemic and necrotic, and then extend the survival of patients [4–7]. But recent studies have revealed that blocking the blood supply would cause residual cancer cells to release the hypoxia inducible factor and elevate the VEGF expression level, and the state of hypoxia and ischemia would accelerate the frequency of neovascularization, greatly improving the chance of PLC recurrence and affecting the long-term prognosis of patients [3, 8, 9]. In order to improve the application effect of interventional treatment, other target therapeutic measures should be adopted at the same time. Sorafenib, a novel multimolecular targeted therapeutic drug, is currently available in China, which can reduce VEGF expression, regulate the Mcl-1 protein level, and then inhibit tumor growth. In addition, some studies have shown that sorafenib can reverse the immunosuppression of hepatocellular carcinoma and improve the immunosenescence condition of patients [10], with a more comprehensive effect.

At present, there have been studies combining interventional treatment with sorafenib in academia, but mostly focusing on the survival rate of patients with few explorations on other aspects. This study aimed to explore the enhancement of sorafenib on the efficacy of interventional treatment comprehensively, with the results reported as follows.

## 2. Materials and Methods

### 2.1. General Information

120 PLC patients admitted to The Second People's Hospital of Dongying from January 2016 to January 2020 were selected as the research object and divided into group A and group B according to the admission order, with 60 cases each and no statistical difference in their general information (*p* > 0.05), as given in Table 1. The study was approved by the Ethics Committee of The Second People's Hospital of Dongying.

### 2.2. Inclusion Criteria

The inclusion criteria of the study were as follows. (1) The patients or their family members fully understood the study process and signed the informed consent; (2) the patients were diagnosed with PLC by clinical and pathological examinations and met the diagnose criteria in the Primary Liver Cancer Diagnose and Treatment Standard (2011 Ver.) [11]; (3) the expected survival time of the patients was over 3 months, and their clinical materials were complete; (4) the patients were at least 18 years old; (5) the liver function class of the patients was A or B [12]; (6) the Karnofsky (KPS) scores of the patients were over 60 points [13]; (7) the follow-up visit was acceptable to the patients; and (8) the patients did not meet the surgical indication and were required to undergo interventional treatment [14].

### 2.3. Exclusion Criteria

The exclusion criteria for the patients of the study were as follows. (1) Presence of mental problems or inability to communicate with others; (2) suffering from other organic diseases, coagulation disorders, or second primary tumor; (3) allergy to the drugs involved in the study; (4) in pregnancy or lactation; (5) presence of alimentary tract hemorrhage; and (6) suffering from inferior vena cava thrombus.

### 2.4. Methods

Both groups of patients received interventional therapy, and sorafenib was given to group A additionally, with the following specific steps. (1) Intervention therapy: after routine skin preparation and disinfection, the patients received local anesthesia. The 5-FRH catheter was inserted to the superior mesenteric artery, common hepatic artery, and proper hepatic artery for visualization, and then, the blood supply artery puncture targeted at primary lesions was conducted according to the lesion condition of the patients, and the ultrasmooth guide wire was used to fix the catheter; then, the mixture of 55 mg/m^2^ of oxaliplatin (manufactured: Harbin Pharmaceutical Group Bioengineering Co., Ltd.; NMPA Approval No. H20133094), 40 mg/m^2^ of hydroxyacetophenone (manufactured: Zhejiang Kancheer Pharmaceutical Co., Ltd.; NMPA Approval No. H42021857), 40 mg/m2 of epirubicin (manufactured: Jiangsu Hengrui Medicine Co., Ltd.; NMPA Approval No. H20020542), and ultraliquid iodized oil was administered once every month for 12 weeks. (2) Sorafenib treatment: the patients orally took 0.5 g of sorafenib (manufactured: Jiangxi Shanxiang Pharmaceutical Co., Ltd.; NMPA Approval No. H20203397) twice every day with warm water. A course lasted for two weeks, and after 1 course, administration was stopped for 2 weeks before entering into a new one. The entire treatment lasted for 12 weeks.

Patients in both groups were followed up for 24 months.

### 2.5. Observation Criteria

1. Treatment effect: according to the RECIST (Response Evaluation Criteria in Solid Tumors) of the WHO, patients' conditions were classified as complete response (CR, disappearance of all lesions, no new lesions, and tumor markers returned to normal for a month), partial response (PR, ≥30% decrease of SLD (the sum of the longest diameters) for a month), stable disease (SD, <30% decrease of SLD or <20% increase of SLD), and progressive disease (PD, ≥20% increase of SLD, or new lesions). The objective remission rate (ORR) = CR + PR and the disease control rate (DCR) = CR + PR + SD were used to compare the treatment effect [15].
2. Survival: the one-year survival rate and two-year condition rate of patients were compared between the two groups.
3. Adverse reaction rate (ARR): the adverse reactions included skin reactions, gastrointestinal reactions, hepatorenal reactions, rash, fatigue and drowsiness, and hyperbilirubinemia, and the numbers of patients with adverse reactions were counted.
4. Serum AFP, VEGF, and GGT levels: 5 ml of fasting elbow vein blood was extracted from the patients before treatment and at the 4th week and 12th week of treatment to detect their AFP, VEGF, and GGT levels with the ELISA assay (Beijing Kewei Clinical Diagnostic Reagent Inc.; NMPA Approval No. S20060028).

### 2.6. Statistical Processing

In this study, the data processing software was SPSS20.0, the picture drawing software was GraphPad Prism 7 (GraphPad Software, San Diego, USA), items included were enumeration data and measurement data, methods used were the *X*^2^ test and *t*-test, and differences were considered statistically significant at *p* < 0.05.

## 3. Results

### 3.1. Comparison of the Patients' Treatment Effect

Group A obtained significantly higher ORR and DCR than group B (*p* < 0.05), as given in Table 2.

### 3.2. Comparison of Patients' Survival

**Figure 1:**
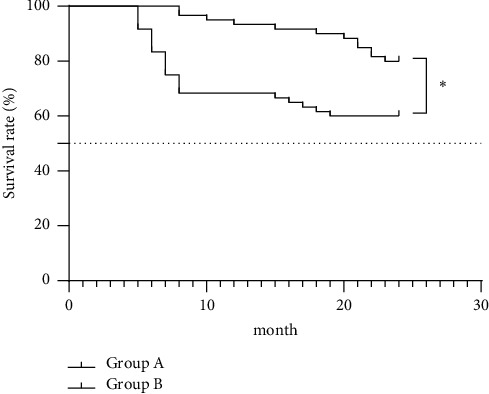
Comparison of patients' survival. The horizontal axis indicates the month, and the vertical axis indicates the survival rate (%). ^*∗*^*P* < 0.05.

Compared with group B, group A obtained significantly higher one-year survival rate (95.0% (57/60) vs. 68.3% (41/60), *p* < 0.05) and two-year survival rate (80.0% (48/60) vs. 60.0% (36/60), *p* < 0.05), as shown in Figure 1.

### 3.3. Comparison of Patients' ARR

**Figure 2:**
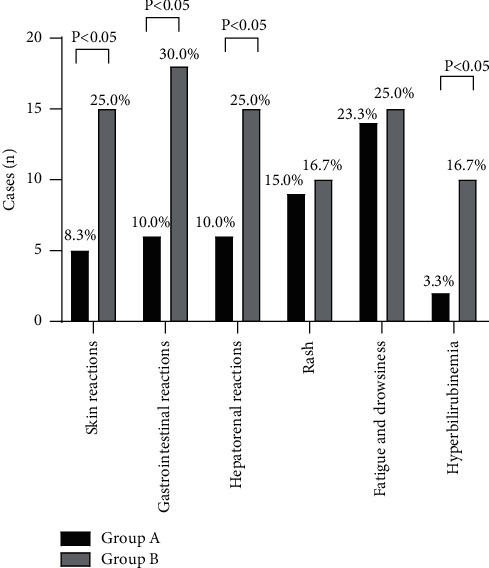
Comparison of patients' ARR (*n* (%)). The horizontal axis from left to right indicates the skin reactions, gastrointestinal reactions, hepatorenal reactions, rash, fatigue and drowsiness, and hyperbilirubinemia, and the vertical axis indicates the cases (*n*); the black areas indicate group A and the gray areas indicate group B.

The ARRs of skin reactions, gastrointestinal reactions, hepatorenal reactions, and hyperbilirubinemia of group A were significantly lower than those of group B (*p* < 0.05), as shown in Figure 2.

No statistical differences were shown in the ARRs of rash and fatigue and drowsiness (9 vs. 10, 14 vs. 15, *p* > 0.05), and the ARRs of skin reactions, gastrointestinal reactions, hepatorenal reactions, and hyperbilirubinemia of group A were significantly lower than those of group B (5 vs. 15, 6 vs. 18, 6 vs. 15, 2 vs. 10, *p* < 0.05).

### 3.4. Comparison of Patients' Serum AFP, VEGF, and GGT Levels

**Figure 3:**
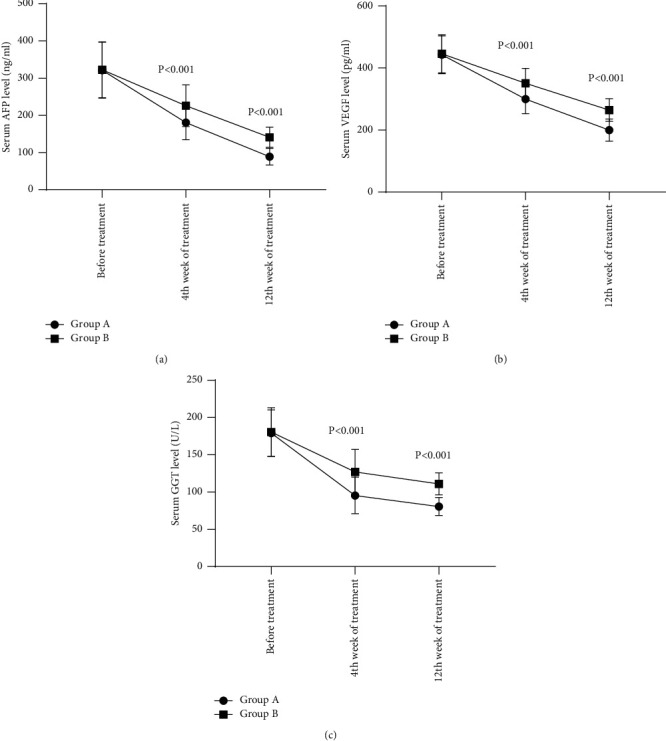
Comparison of patients' AFP, VEGF, and GGT levels ($\bar{x}\pm s$). The horizontal axis from left to right indicates before treatment, 4^th^ week of treatment, and 12^th^ week of treatment; the lines with dots indicate group A and the lines with blocks indicate group B.

The treated serum AFP, VEGF, and GGT levels of group A were significantly lower than those of group B (*p* < 0.001), as shown in Figure 3.

Figure 3(a) shows the serum AFP level. No statistical differences are shown in the serum AFP levels between the two groups before treatment (321.65 ± 75.26 vs. 322.69 ± 74.98, *p* > 0.05); at 4^th^ week and 12^th^ week of treatment, group A obtained significantly lower serum AFP levels than group B (180.65 ± 45.98 vs. 225.99 ± 55.98 and 88.95 ± 22.62 vs. 140.95 ± 26.98, *p* < 0.001).

Figure 3(b) shows the serum VEGF level. No statistical differences are shown in the serum VEGF levels between the two groups before treatment (442.56 ± 60.89 vs. 445.66 ± 61.55, *p* > 0.05); at 4^th^ week and 12^th^ week of treatment, group A obtained significantly lower serum VEGF levels than group B (299.65 ± 46.98 vs. 350.98 ± 47.99 and 199.98 ± 35.87 vs. 264.48 ± 36.98, *p* < 0.001).

Figure 3(c) shows the serum GGT level. No statistical differences are shown in the serum GGT levels between the two groups before treatment (178.98 ± 31.25 vs. 180.56 ± 32.65, *p* > 0.05); at 4^th^ week and 12^th^ week of treatment, group A obtained significantly lower serum GGT levels than group B (95.41 ± 24.56 vs. 126.98 ± 30.11 and 80.54 ± 12.10 vs. 110.98 ± 14.65, *p* < 0.001).

## 4. Discussion

PLC has an insidious onset, and the patients who are diagnosed with PLC are mostly in the middle and late stages of the disease, so they can only be treated with local nonsurgical modalities to prolong survival rather than radical surgery. 90% of PLCs are hepatocellular carcinomas (HCC), and the blood supply of HCC is mainly from the hepatic artery, and only a small amount is from the portal vein; in contrast to the blood supply ratio of normal liver tissue, the interventional therapy can target the blocking of the hepatic artery supply, so that the tumor tissue loses its blood supply source without affecting normal liver tissue, which is the preferred nonsurgical treatment option for patients [16–18]. Notably, interventional therapy, although it can substantially prolong the survival time of patients, cannot eliminate all cancer cells, and residual cells in hypoxic and ischemic conditions elevate the expression of the hypoxia inducible factor, which can increase VEGF transcription and then accelerate the frequency of neoangiogenesis, so that patients are prone to recurrence and metastasis of liver cancer. Therefore, combining other target therapeutic measures with the interventional therapy to optimize the long-term outcome is the key to lower the mortality in PLC patients [19].

Molecular targeted therapy is an important way to inhibit the growth of cancer cells, which can act in the links of cancer cell survival, growth, or neoangiogenesis with the toxicity significantly lower than chemotherapy, thus playing a specific antitumor role and being able to combine with different interventional therapies. Sorafenib, a novel molecular targeted therapeutic agent, can block the signal pathway of receptor tyrosine kinase, reduce VEGF activity in the hepatic artery, slow down the neoangiogenesis for oxygen and blood supply, and thus inhibit cancer cell metastasis [20, 21], so the treated VEGF level of patients in group A who received both treatment modalities was significantly lower than that in group B (*p* < 0.001), indicating a reduced migration rate of the cancer tissue.

In addition to receptor tyrosine kinase, there are several other receptors for sorafenib, which can suppress multiple protein expression levels of cell proliferation through other pathways, alleviate cytotoxicity, and accelerate the repair process of liver function. In this study, GGT was selected as an indicator of liver function, which mostly presented in the human liver and was able to monitor hepatobiliary disease. The GGT level in PLC patients can be over dozens of times higher than the normal person, and the decrease of GGT directly indicates the improvement of liver function in patients and indirectly suggests the reduction of liver cancer cells [22]. The result showed that group A achieved significantly lower GGT level than group B (*p* < 0.001), reflecting that recovery was seen in the patients' liver function and the likelihood of experiencing liver toxic side effects decreased concomitantly. Moreover, Pereira et al.' study confirmed that sorafenib could also induce the movement of lymphocytes to HCC tissues and reverse the immunosuppression of HCC [23], while Meizhen et al.' study showed that this drug could downregulate AFP expression and relieve immunosuppression in hepatitis B patients [24]. AFP is a class of protein with immunosuppression that can reduce the proliferation rate of T lymphocytes and inhibit the activity of tumor cytokines. This study showed that AFP levels after treatment in both groups were reduced because the intervention could accelerate the frequency of cancer cell necrosis and scavenge immunosuppressive factors, thus improving the immune function of patients, and group A obtained obviously lower AFP levels than group B (*p* < 0.001), confirming that the combined therapy could achieve a better immune recovery effect and enhance the patient body resistance, which further lowered the ARR and comprehensively promoted the overall efficacy of PLC patients. Therefore, the ORR, DCR, and survival rate of group A were significantly higher than those of group B (*p* < 0.05), indicating that the combined therapy obtained a remarkably better effect than intervention alone in terms of long-term outcomes.

To sum up, sorafenib combined with interventional therapy can inhibit PLC, enhance the body immunity, and prolong the survival time of patients, which should be promoted in practice.

## Figures and Tables

**Table 1:** Comparison of patients' general information.

Group	Group A (*n* = 60)	Group B (*n* = 60)	*X*^2^/*t*	*P*
Gender				
Male	52	50	0.261	0.609
Female	8	10		

Age (years)				
Range	34–76	35–76		
Mean age	46.32 ± 5.21	46.98 ± 5.25	0.691	0.491

Liver function class				
A	48	47	0.051	0.822
B	12	13		

Hepatitis type				
Hepatitis C	10	12	0.223	0.637
Hepatitis B	50	48		

Complication				
Ascites	30	32	0.134	0.715
Portal vein tumor thrombus	10	9	0.063	0.803
Cirrhosis	42	40	0.154	0.695

TNM staging				
II	24	25	0.035	0.853
III	26	25	0.034	0.853
IV	10	10	0.000	1.000

Clinical type				
Diffused	10	12	0.223	0.637
Nodal	20	21	0.037	0.847
Massive	30	27	0.301	0.583

Maximum diameter of tumor (mm)				
Range	50–140	48–142		
Mean diameter	85.65 ± 20.65	86.98 ± 20.66	0.353	0.725

ECOG score^*∗*^				
0 point	35	36	0.035	0.853
1 point	25	24		

Educational degree				
Senior high school and below	22	21	0.036	0.849
College and above	38	39		

^*∗*^The physical health and therapy resistance of patients.

**Table 2:** Comparison of patients' overall efficacy (*n* (%)).

Group	CR	PR	SD	PD	ORR	DCR
Group A	24 (40.0)	30 (50.0)	2 (3.3)	4 (6.7)	54 (90.0)	56 (93.3)
Group B	18 (30.0)	20 (33.3)	10 (16.7)	12 (20.0)	38 (63.3)	48 (80.0)
*X* ^2^	1.319	3.429	5.926	4.615	11.926	4.615
*P*	0.251	0.064	0.015	0.032	0.001	0.032

## Data Availability

The data used to support the findings of this study are available from the corresponding author upon reasonable request.
